# Addressing image misalignments in multi-parametric prostate MRI for enhanced computer-aided diagnosis of prostate cancer

**DOI:** 10.1038/s41598-023-46747-z

**Published:** 2023-11-13

**Authors:** Balint Kovacs, Nils Netzer, Michael Baumgartner, Adrian Schrader, Fabian Isensee, Cedric Weißer, Ivo Wolf, Magdalena Görtz, Paul F. Jaeger, Victoria Schütz, Ralf Floca, Regula Gnirs, Albrecht Stenzinger, Markus Hohenfellner, Heinz-Peter Schlemmer, David Bonekamp, Klaus H. Maier-Hein

**Affiliations:** 1 Division of Medical Image Computing, German Cancer Research Center (DKFZ) Heidelberg, Im Neuenheimer Feld 223, 69120 Heidelberg, Germany; 2 Division of Radiology, German Cancer Research Center (DKFZ) Heidelberg, Heidelberg, Germany; 3 Medical Faculty Heidelberg, Heidelberg University, Heidelberg, Germany; 4 Helmholtz Imaging, German Cancer Research Center (DKFZ) Heidelberg, Heidelberg, Germany; 5 Faculty of Mathematics and Computer Science, Heidelberg University, Heidelberg, Germany; 6 Mannheim University of Applied Sciences, Mannheim, Germany; 7 Junior Clinical Cooperation Unit ‘Multiparametric Methods for Early Detection of Prostate Cancer’, German Cancer Research Center (DKFZ) Heidelberg, Heidelberg, Germany; 8 Department of Urology, University of Heidelberg Medical Center, Heidelberg, Germany; 9 Interactive Machine Learning Group, German Cancer Research Center (DKFZ) Heidelberg, Heidelberg, Germany; 10 Institute of Pathology, University of Heidelberg Medical Center, Heidelberg, Germany; 11 German Cancer Consortium (DKTK), DKFZ, Core Center Heidelberg, Heidelberg, Germany; 12 Pattern Analysis and Learning Group, Department of Radiation Oncology, Heidelberg University Hospital, Heidelberg, Germany

**Keywords:** Magnetic resonance imaging, Prostate cancer, Biomedical engineering, Software

## Abstract

Prostate cancer (PCa) diagnosis on multi-parametric magnetic resonance images (MRI) requires radiologists with a high level of expertise. Misalignments between the MRI sequences can be caused by patient movement, elastic soft-tissue deformations, and imaging artifacts. They further increase the complexity of the task prompting radiologists to interpret the images. Recently, computer-aided diagnosis (CAD) tools have demonstrated potential for PCa diagnosis typically relying on complex co-registration of the input modalities. However, there is no consensus among research groups on whether CAD systems profit from using registration. Furthermore, alternative strategies to handle multi-modal misalignments have not been explored so far. Our study introduces and compares different strategies to cope with image misalignments and evaluates them regarding to their direct effect on diagnostic accuracy of PCa. In addition to established registration algorithms, we propose ‘misalignment augmentation’ as a concept to increase CAD robustness. As the results demonstrate, misalignment augmentations can not only compensate for a complete lack of registration, but if used in conjunction with registration, also improve the overall performance on an independent test set.

## Introduction

Diagnosis of prostate cancer (PCa) is one of the most challenging tasks in oncology due to its complex diagnostic chain^1–4^. Magnetic resonance imaging (MRI) is quickly becoming the standard of care for pre-biopsy evaluation to determine whether targets are present for trans-rectal ultrasound-guided stereotactic fusion biopsy. MRI interpretation has been standardized by the Prostate Imaging Reporting and Data System (PI-RADS)^5–7^, currently in version 2.1^8^. According to PI-RADS, standard prostate MRI is acquired in a multi-parametric (mp) fashion, including T2-weighted (T2w), diffusion-weighted (DWI) and dynamic contrast-enhanced (DCE) sequences. Regarding localized PCa, the MRI index lesion, which is the most suspicious lesion according to the zone-specific MRI appearance, dictates the overall PI-RADS score. The patient-based assessment both for PI-RADS in terms of the highest PI-RADS score of any lesion and the whole-prostate highest Gleason Grade Group histopathological assessment, convey the most important clinical information for treatment decisions^7^.

While PI-RADS has led to better standardization, its inter-rater variability remains high^9,10^ and interpreting the MRI images requires radiologists with a high level of task-specific expertise^11^. Thus, there is an increasing interest in clinically applicable computer-aided diagnosis (CAD) tools to support radiologists in prostate MRI decision making, and especially in convolutional neural network (CNN) based systems^12,13^ due to their recent successful application to complex clinical problems^14–16^. Due to its inherent interpretability and clinical value, approaches based on semantic segmentation are the most popular image analysis task in the biomedical community, which have already been successfully applied to the diagnosis and intervention planning of PCa^17–24^. Semantic segmentation has become even more applicable due to the self-configuring framework of nnU-Net^25^ for medical images. It allows a standardized state-of-the-art performance and it has already been successfully adopted for patient-level PCa diagnosis^23,26^.

Though CAD systems achieve outstanding performances, there is no common consensus on how to handle misalignments that occur between acquisitions. Despite instructions for patients to remain still during acquisition that typically lasts 30 minutes or more, slight movements can still lead to misalignments between sequences. Even in shorter time frames, soft tissue deformation may occur due to involuntary muscle contractions e.g. of the bowel or slow bladder filling, resulting in local elastic misalignments^27^. Additionally, each MRI sequence, in other words modality, responds differently to prostate tissue properties, leading to a unique contrast characteristic and thus in different lesion contours. Importantly, geometric distortions resulting from susceptibility effects can lead to different positioning of tissue depending on the type of imaging sequence^27^, especially between the T2w and DWI sequences^28–30^. These misalignments can result in prostate lesions being notably misaligned between modalities, and thereby performing clinical segmentations on one modality does not ensure accurate lesion ground truth segmentation on the other modalities. Consequently, this can potentially limit model performance, particularly in applications that rely on semantic segmentation being sensitive to spatial information.

Co-registration of the input image modalities is usually performed as a pre-processing step to eliminate misalignments between the sequences, thereby matching the ground truth segmentations to all modalities^31^. Some research groups have implemented complex deformable registration algorithms trying to align the anatomical structures on top of each other correcting local elastic misalignments additionally to the global misalignments^26,32^. To avoid implausible registration transformations, of which risk is especially present in local regions around the prostate gland where the similarity metric fails due to image artifacts like rectal gas-induced artifacts^27–29^, many studies limit their registration to non-elastic deformation fields^17,22,33^. Other research studies^23,24,34^ claim that standardized precautionary measures (namely minimal temporal difference between acquisitions, administration of antispasmodic agents to reduce bowel motility, use of rectal catheter to minimize distension^27,35,36^) are sufficient to eliminate the need for registration^23^ supporting these claims with visual observations.

Although the publications above presented valuable insights, the influence of registration on the diagnostic performance of CAD systems remains unexplored. Studies that additionally applied non-rigid transformations also did not investigate whether their downstream task benefited from that complex registration or not. It is known that registration methods are seldomly perfect, particularly in the case of prostate MRI, with its often anisotropic voxel dimensions required to guarantee sufficient signal-to-noise ratio and with the common deformations especially occurring on the susceptible diffusion sequences. Consequently, misalignments may remain and could cause issues in CNN training and inference. Furthermore, the use of alternative strategies to handle multi-modal misalignments in PCa diagnosis has not been studied so far.

In this work we are investigating multiple strategies for dealing with misalignments for enhanced PCa diagnosis. Importantly, we base our conclusions on the performance of the clinical downstream task of patient-level PCa diagnosis derived from the CAD system as opposed to surrogate alignment or similarity measures. Our results do not only underline the importance of registration, but we propose to tackle the problem of misalignments and remaining registration errors by introducing the new strategy of misalignment augmentation, a data augmentation technique that simulates additional, probabilistic registration errors on-the-fly during training. By substantially augmenting the diversity of misalignments in the training data, the hope is to teach CNNs to become robust and invariant to them to some extent, thus increasing their performance on unknown datasets. Finally, we investigate the complementary value of misalignment augmentation in combination with registration, demonstrating improvements that go beyond pure registration-based techniques.

## Results

We evaluated different strategies to deal with misalignments on the clinical task of patient-level PCa diagnosis opposed to surrogate alignment or similarity measures. As the current state-of-the-art method for PCa diagnosis, we derived the diagnosis through semantic segmentation of malignant lesions. Given that semantic segmentation directly depends on spatial information, it was particularly well suited for testing strategies for dealing with misalignments. We evaluated two strategies with different objectives:

- we co-register the MRI sequences using B-spline registration to eliminate misalignments and to match the ground truth segmentations with all image modalities,
- we propose misalignment augmentation, a new strategy to make CNNs robust for misalignments and to tackle remaining registration errors.

Our evaluation approach aimed to disconnect the assessment of strategies for handling misalignments from surrogate alignment measures and focus on their performance in the clinical task. We supported this approach by investigating the relationship between lesion-wise Dice score density functions and patient-wise AUROC values for different registration techniques.

We summarize the results of our systematic analysis of the effect of B-spline registration and misalignment augmentation on an independent multicentric test set consisting of 129 bi-parametric MRI (bpMRI) exams with biopsy-confirmed diagnosis. The following results not only show the importance of registration, but also underline the importance of misalignment augmentation due to its induced high regularization effect. Furthermore, the results reveal their complementary effect.

### Achieving the most robust setup by combining registration and misalignment augmentation

To find out the most robust setup against multi-modal image misalignments, the effects of registration and misalignment augmentation are systematically evaluated on the patient-level area under the receiver operating characteristic curve (AUROC). The results are summarized in Table 1.

**Table 1:** Patient-level AUROC results with 95% confidence intervals (CI) and p-values resulted from the DeLong test on the independent test set. Addressing misalignments either with registration or with misalignment augmentation increased the AUROC value compared to the unregistered dataset without misalignment augmentation. The highest AUROC value with statistically significant improvement is reached by combining registration and misalignment augmentation.

AUROC (test set)	Unregistered dataset	Registered dataset
Without misalignment augmentation	75.93% (CI: 67.49–83.68, reference)	79.11% (CI: 70.95–86.93, $p=0.31$)
With misalignment augmentation	80.13% (CI: 71.57–87.18, $p=0.11$)	82.07% (CI: 74.18–89.38, $p=0.02$)

Addressing misalignments either with registration or with misalignment augmentation increased the AUROC value compared to the unregistered dataset, but neither of these improvements reached statistical significance, with p-values of $p=0.31$ and $p=0.11$, respectively. However, combining these strategies reached the highest AUROC value with statistically significant improvement ($p=0.02$) compared to the unregistered dataset without misalignment augmentation, indicating that this is the most robust setting addressing misalignments.

Furthermore, we evaluated individually our methods on each dataset included for this study (PROSTATEx and in-house dataset) and found that the AUROC values were consistent, indicating that our approach is effective across the two data centers. The AUROC results for each dataset can be found in ‘Supplementary Table S4’.

The test results are presented in more detail in the following subsections.

### Predictive performance competitive with radiologists

**Figure 1:**
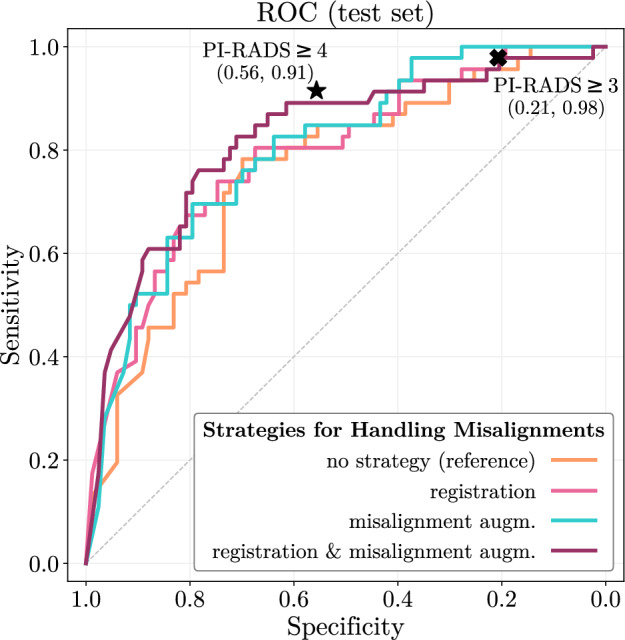
Predictive performance comparison of the trained models and the radiologists on the ROC. The radiologists’ performance with PI-RADS $\ge$ 3 and $\ge$ 4 are marked to locate clinically informative performance points. The sensitivity in the low and high specificity area is increased using either registration or misalignment augmentation compared to the unregistered dataset. Their performance closely match and slightly exceed the PI-RADS $\ge$ 3 point, respectively, but none of them improved the sensitivity towards PI-RADS $\ge$ 4. Combining registration and misalignment augmentation closely matches both PI-RADS performance points with the highest sensitivity improvement over the widest specificity range.

To highlight the practical advantages of techniques addressing misalignments, we compare the performance of the trained models to radiologists’ interpretation on our cohort. We calculated the ROC curves for every model and the radiologists’ performance for PI-RADS $\ge$ 3 and $\ge$ 4, which locate clinically informative performance points on the ROC diagram. Figure 1 illustrates the effect of registration, misalignment augmentation and their combination on the ROC. The radiologists’ performance with PI-RADS $\ge$ 3 and $\ge$ 4 with specificity and sensitivity of $\left( 0.21, 0.98\right)$ and $\left( 0.56, 0.91\right)$, respectively, are marked in Fig. 1 too.

Using either registration or misalignment augmentation increased the sensitivity in the low and high specificity area compared to the unregistered dataset without misalignment augmentation. The performance of the registered dataset closely match, the unregistered dataset with misalignment augmentation sightly exceed the PI-RADS $\ge$ 3 point, but none of them increased the sensitivity towards PI-RADS $\ge$ 4. However, their combination was the only setting, which closely matches both clinical PI-RADS performance points and increased the sensitivity over the widest specificity range.

### Qualitative results support our findings

**Figure 2:**
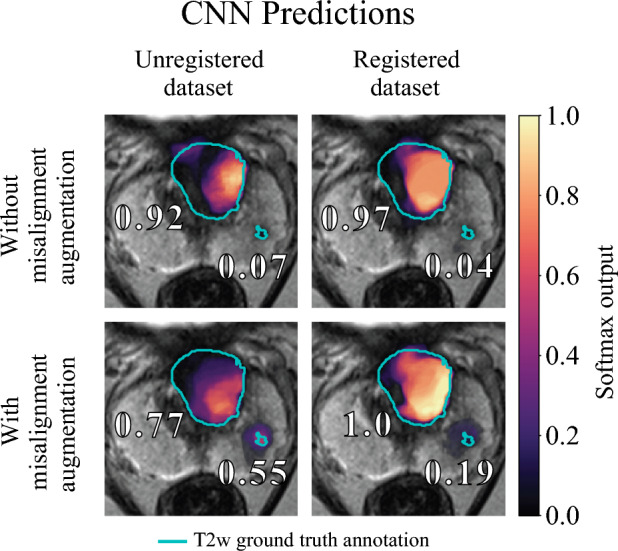
Comparison of manual annotations and segmentation predictions for a patient exam with one large lesion (PI-RADS 4, GS 7a) and with one puncture lesion (PI-RADS 4, GS 7a) in the left prostate base. On a representative T2w slice of the index lesion manual annotations given as a cyan outline as well as the probability map from the different settings superimposed using a colormap, with the transparency of probability values raised to the power of 0.2. CNN probabilities are given in the first row for the unregistered dataset and for the registered CAD dataset. The bottom row shows the same datasets in the first row with misalignment augmentation. The highest probability values for the individual lesions are marked lesion-wise too. All of the CNNs predicted the large index lesion with high confidence. However, the highest predictions for the second punctate lesion belong to settings with misalignment augmentations.

To provide a short visual insight, the influence of registration, misalignment augmentation and their combination on the predicted segmentation results are visualized. Figure 2 shows the manual annotations as well as the lesions identified by semantic segmentation in terms of a color scale for a patient exam with one large lesion (PI-RADS 4, GS 7a) and with one puncture lesion (PI-RADS 4, GS 7a) in the left prostate base.

Registration and misalignment augmentation increased the predicted lesion volume for both datasets resulted in a more complete coverage of the pathological lesions. Additionally, misalignment augmentation enabled segmentation of a small punctate lesion in the PZ too, which was not picked up by the other configurations.

### Proposed method on par with method that uses ground-truth human segmentation

**Figure 3:**
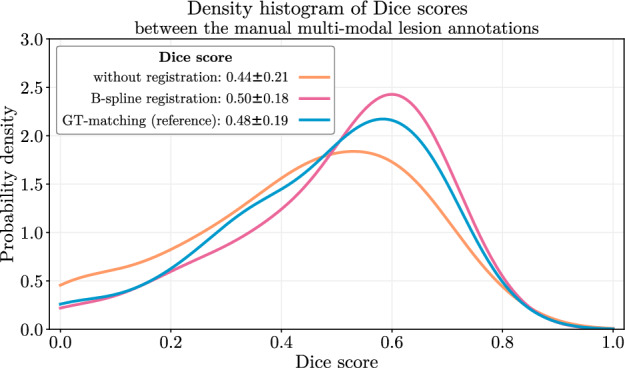
Dice score probability density functions between the manual lesion annotations in the T2w and ADC modalities for our dataset without registration, with B-spline registration, and with the reference GT-matching. Mean and standard deviation values are calculated for all settings and located in the corresponding legend entries. The best Dice score distribution belongs to the B-spline registration sightly outperforming the reference GT-matching.

To support the quality of the used B-spline registration method for our CAD system, we compare it with the reference ground-truth-matching (GT-matching) registration. For that, a lesion overlap metric and ROC of the actual clinical task were used. Figure 3 shows the Dice score probability density functions for our dataset using different registration techniques, as well as without any registration.

**Figure 4:**
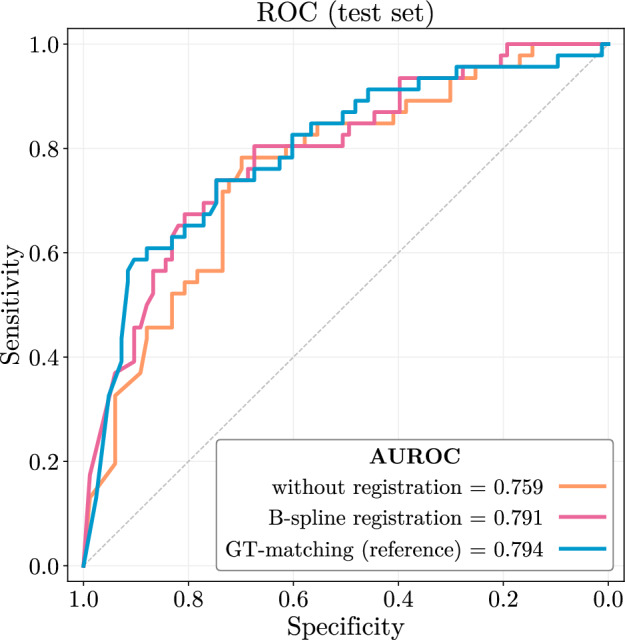
The influence of the different registration techniques on the predictive performance. Using B-spline registration and the reference GT-matching, increased the AUROC value of the unregistered dataset by 3.2% and 3.5%, respectively, especially the sensitivity in the low and high specificity ranges.

The mean and standard deviation of the Dice score for the unregistered dataset of $0.44\pm 0.21$ is improved to $0.48\pm 0.19$ using the GT-matching and to $0.50\pm 0.18$ using the B-spline registration slightly outperforming the reference. The corresponding ROC curves are illustrated in Fig. 4.

Both registration techniques, B-spline registration and GT-matching, increased the AUROC value by 3.2% and 3.5%, respectively, compared to the unregistered dataset. However, the differences in the Dice score density functions between the two registered datasets did not translate to the differences in the corresponding AUROC performances.

## Discussion

This paper systematically evaluates and highlights the importance of handling multi-modal image misalignments both by registration and our proposed misalignment augmentation for enhanced patient-level prostate cancer diagnosis on a multicentric prostate bpMRI dataset.

Registering the MRI sequences increased the patient-level AUROC compared to the unregistered dataset. The results indicate that aligning the anatomical structures on top of each other helped the CAD system to couple multi-modal information, which leads to improved downstream performance. Furthermore, the lack of the performance difference in the AUROC values of the different registration techniques compared to the observable differences in the Dice score density functions indicates that the surrogate measures for evaluating registration performance do not necessarily translate to the clinical downstream task performance. This underlines the importance of our study evaluating the quality of registration on the clinical task of patient-level PCa diagnosis. Despite the observed improvement in the AUROC value resulting from registering the MRI sequences, the improvement was not statistically significant.

Generating augmented misalignments between the modalities during the training of CNNs made the diagnostic task harder and forced the network to become more invariant for misalignments. We showed that using these augmentations on the unregistered dataset could replace registration due to its induced regularization effect. Thus, misalignment augmentations have been shown to be able to address image misalignments and potentially simplify preprocessing by replacing complex non-rigid registration techniques in cases, where the anatomical structures are already partially overlapped. It is important to note that the ability of the method to cope with misalignments is limited depending on the initial overlap between the image modalities, and the type and amplitude of the augmentations. Thus, its parameters have to be adapted to the exact clinical need, and an initial registration is still needed. However, the improvement was not statistically significant, similarly to registration.

Although we addressed the same problem with registration and misalignment augmentation their combination resulted in further improvement in the ROC with a statistically significant difference compared to the unregistered dataset. A potential explanation for this complementary behavior and for the statistically significant improvement could be that these strategies extended each other’s limitations. While registration provides anatomical matching for learning more complex features, misalignment augmentation could further increase the robustness of the CNNs against remaining registration errors. It is worth mentioning that in the case of larger datasets, the model’s capability to autonomously learn to cope with misalignments without assistance remains an open question. The inherent size and diversity of the data might enable the model to naturally adjust to the broad spectrum of misalignments to some extent. However, in the context of medical datasets with limited size, registration and in particular augmentation techniques, like misalignment augmentation play a vital role in inducing application-specific knowledge, effectively enhancing the model’s ability to adapt to various misalignments and maintain robust performance. This configuration not only reached the highest AUROC value on the test set, but it also pushed the ROC curve closer to the radiologists’ performance closely matching PI-RADS $\ge$ 4, which was not reached by any other configurations. Our experiments indicate that combining registration with misalignment augmentation is the optimal configuration for the training of multi-modal prostate MRI datasets. Furthermore, our results suggest integrating the paradigm of misalignment augmentation into the standard repertoire of CNN design for prostate MRI studies and utilizing this data augmentation as a blueprint for other multi-modal applications.

## Methods

### Prostate MRI cohort

We included 625 examinations from two cohorts in this study: 204 exams from the publicly available PROSTATEx^37^ challenge dataset and an in-house cohort consisting of 421 consecutive examinations acquired during clinical routine from 2014 to 2016. Exams from the in-house cohort were previously published and included in this study following the same inclusion criteria as in^18,19,26,38^. The ethics committee of the Medical Faculty Heidelberg approved the study and waived informed consent (institutional ethics approval number S-164/2019) to enable analysis of a consecutive cohort. All experiments were performed in accordance with the declaration of Helsinki (64th WMA General Assembly, Fortaleza, Brazil, October 2013) and relevant data privacy regulations. More details about our cohort can be seen in ‘Supplementary Tables S1 and S2’.

Although the PI-RADS manual suggests mpMRI including DCE for prostate MRI assessment, bi-parametric MRI (bpMRI) abbreviated protocols, consisting only of T2-weighted imaging (T2w) and DWIs, are being discussed to be of acceptable diagnostic quality. bpMRI may allow a minimal trade-off in diagnostic quality at the advantage of sparing contrast agent administration and gaining faster image acquisition^39^, properties which also have made bpMRI an attractive design choice for promising pioneering deep learning^40,41^ applications in prostate MRI. Therefore, T2w, DWIs with high b-value, and ADC maps were used for this study.

For all patients, PI-RADS (either v1, v2^5^ or v2.1^8^) interpretation was performed by board-certified radiologists during clinical routine. Where only PI-RADS v1 was available in the clinical report, a retrospective PI-RADS v2 read was performed by a board-certified radiologist. These clinical reports are used for the lesion annotation process and the evaluation of the human performance.

Based on the clinical reports, the lesions of the in-house cohort were segmented retrospectively on both modalities by multiple in-house investigators using the Medical Imaging Interaction Toolkit (MITK)^42,43^ under the supervision of a board-certified radiologist with more than 13 years of experience in prostate MRI interpretation (D.B.), as previously described in^19,26,38^. Lesions in the PROSTATEx training dataset were manually segmented by the same investigators using the publicly provided lesion coordinates. The lesion segmentations were used as the ground truth for the semantic segmentation task. Additionally, in a subset of the cohort the prostate gland was also segmented manually, then prostate segmentation CNNs trained and segmentation proposals generated for the remainder of the examinations, which were reviewed and manually edited. The prostate masks were not only used for the co-registration of the modalities for creating a reference dataset (see later in section “Registration methods and annotations for training”), but also for selecting the clinically relevant lesions by combining the different biopsy approaches, see later in this section.

In-house patients underwent MRI trans-rectal ultrasound-fusion transperineal biopsy using real-time registration between the T2w and ultrasound images. The evaluation of the histopathological samples was performed according to the International Society of Urological Pathology (ISUP) standards under the supervision of a dedicated uropathologist with more than 19 years of experience (A.S.). Clinically significant prostate cancer (csPCa) was defined as ISUP grade 2 or higher^44,45^. Findings for the PROSTATEx cohort are also biopsy confirmed where csPCa was defined by Gleason score (GS) 7 or higher.

The patient-wise ground truth is determined as the maximum ISUP grade of all available biopsies taken during the biopsy session based one of the MRI examinations for the in-house dataset, or from Gleason score provided as ground truth for the PROSTATEx challenge. The in-house dataset demonstrates 34.44% and the PROSTATEx dataset demonstrates 34.31% prevalence for csPCa. The exact distribution can be seen in Table 2. The patient-wise ground truth was used for the patient-wise evaluation (see section “Evaluation of the clinical downstream task”).

**Table 2:** Patient distribution through the datasets with respect to csPCa. The datasets are highly imbalanced, which is handled with a balanced trainer (see section “Training protocol”).

Exams	Without csPCa	Without csPCa	Sum
PROSTATEx	134 (65.7%)	70 (34.3%)	204
In-house dataset	276 (65.6%)	145 (34.4%)	421
Sum of exams	410 (65.6%)	215 (34.4%)	625

For every lesion in the in-house dataset, the maximum ISUP grade is determined from a systematic biopsy-enhanced lesion histopathological ground truth (SELGT) that is determined from a histopathological sextant mapping integrating targeted and systematic biopsies according to the Ginsburg protocol^19,46^. This extended biopsy scheme ascertains that most of the csPCa is correctly diagnosed, as previously shown by comparison of this biopsy scheme to radical prostatectomy specimen^47,48^. In PROSTATEx, the Gleason scores were available for every lesion. The lesion-wise ground truth is used to generate the masks for the training, only lesions containing csPCa were selected.

### Registration methods and annotations for training

**Figure 5:**
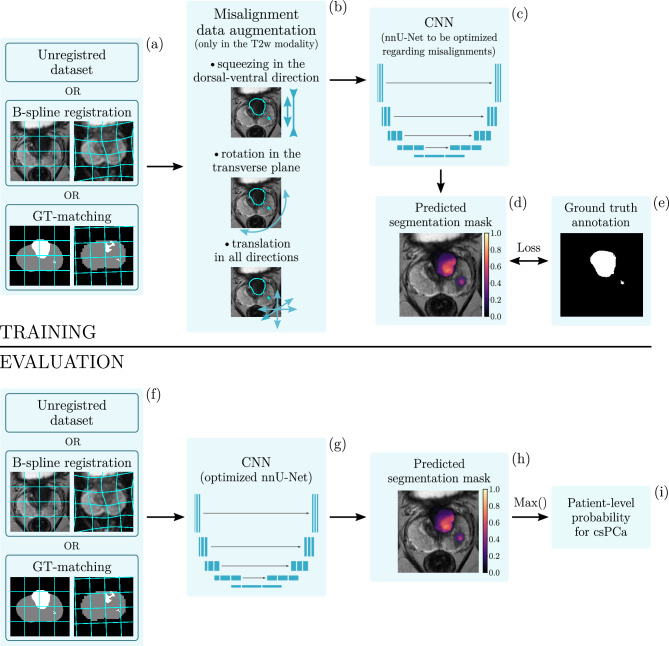
Overview of the training procedure with misalignment augmentation and the evaluation of the trained CNN. Training: (**a**) Three multi-modal datasets with different registration methods were created: an unregistered dataset as a lower bound reference, a dataset using B-spline registration, and a reference GT-matched dataset using the ground-truth segmentations. (**b**) Before any global image transformation, an optional misalignment data augmentation is implemented generating additional plausible misalignments between the image modalities. (**c**–**e**) We optimized the nnU-Net framework with the proposed modifications for the training of Netzer et al.^26^, which was already successfully applied on the same data source. Application: (**f**) The GT-matched dataset is used only as an additional reference dataset for the evaluation of our B-spline registration. (**g**–**i**) Following the same evaluation scheme as Netzer et al.^26^, the patient-wise PCa probability is calculated by taking the maximum value of the predicted lesion masks by the optimized CNN.

To enable methodological comparison, from the mentioned primary prostate MRI cohort (see section “Prostate MRI cohort”), we generated several multi-modal datasets resulting from different image registration techniques (see Fig. 5a,f):

1. We used an unregistered multi-modal dataset with all modalities (T2-w, DWI with the highest b-value, ADC), which we hypothesize to be the lower performance bound in our study.
2. We created a dataset using B-spline registration the same way as Netzer et al.^26^, where the registration is based on voxel intensities using mutual information^49^ as the similarity metric. As the DWIs with the lowest b-values are the most similar to the original T2w images, it was beneficial to use them to calculate the transformation parameters for the co-registration of the two modalities. These parameters are then applied to the DWIs with the highest b-values and the ADC maps. This registration technique enables to interpret the prediction of the CAD on both modalities without any post processing. We use this dataset for our main experiments.
3. We also created as a reference dataset a segmentation based registration similarly to Sanyal et al.^17^ being sure that the segmentations of the lesions and the prostate in the modalities overlap each other. By correcting the inconsistent ground-truths between the modalities resulted by misalignments, we provide a steady reference performance to be able to get information about the quality of our B-spline registration. We call this registration technique the ‘*ground-truth-matching*’ (GT-matching).

Lesion segmentation masks belonging to the T2w modalities were used due to their higher resolution compared to the ADC maps and because they are annotated by also taking information from the ADC maps into consideration.

### Misalignment augmentation

Data augmentation is intended to be a solution for the problem of overfitting by enhancing the size of the training dataset^50,51^. It also helps CNNs to cope with transformations, for which they are not invariant and which occur in the natural distribution of the data, such as rotation and scaling. This is essentially a way of introducing inductive biases into the model training to improve performance and robustness. Misalignments between image modalities could be addressed by using data augmentation as well. To become invariant to them to some extent, we introduce misalignment augmentation, a data augmentation technique generating additional, probabilistic misalignments on the fly during training and which can be dropped into any augmentation pipeline. Here we simply add misalignment augmentation to the standard nnU-Net data augmentation pipeline (see Fig. 5b). The amplitude of each transformation is sampled with uniform distribution constrained by a maximum amplitude value in positive and negative directions following the batchgenerators framework^52^. The three applied transformations for generating misalignments are:

- translations with the maximal amplitudes of $\left( 10, 10, 6\right) \text {mm}$ into the x,y,z directions, respectively,
- rotations with the maximal amplitude of 15$^\circ$ only in the x-y plane due to the highly anisotropic spacing,
- and a small amount of affine squeezing with the maximal ratio of 0.1 only in the dorsal-ventral direction which is similar to the naturally occurring image distortion between the T2-w and DWIs due to magnetic field inhomogeneities^53^.

We make our proposed misalignment augmentations publicly available as part of the batchgenerators framework^52^ https://github.com/MIC-DKFZ/batchgenerators and integrate it into a nnU-Net^25^ trainer https://github.com/MIC-DKFZ/misalignment_DA.

Identifying prostate zones are crucial not only in PI-RADS^8^, but also in the segmentation of lesions with neural networks^34,54^. The T2w images contain rich structural information with high resolution compared to the DWI sequences. Therefore, misalignments are generated by displacing the T2w modality with the corresponding T2w lesion annotations relative to the DWI sequences. To minimize transformation artifacts, the misalignment augmentation is implemented before any global data augmentation.

Though misalignments can be significantly reduced by registration, applying misalignment augmentation for the registered datasets could make CNNs more robust for remaining registration errors.

### Evaluation of the clinical downstream task

Despite recent improvements in the field of registration techniques^55,56^, registration depends on surrogate measures, which do not guarantee that high measurement scores translate into improved clinical predictions^57^. In contrast, we evaluate the effects of registration techniques (see section “Registration methods and annotations for training”) and misalignment augmentation (see section “Misalignment augmentation”) on the clinical downstream task: patient-level csPCa diagnosis.

To get quantitative measures about the quality of the registered CAD dataset, the Dice score probability density functions for every datasets are calculated by using the manual lesion annotations from the T2w and ADC modalities. Calculating this surrogate registration overlap metric not only provides information about the quality of the datasets regarding misalignments, but it also gives information about the correlation between the overlap measures and the clinical performances.

We choose patient-wise whole image PCa diagnosis derived from semantic segmentation for the clinical downstream task (see Fig. 5c–e). Their predictions are not just strongly relying on spatial information, but semantic segmentation is clinically interpretable task by providing spatial localization^14–16^. For assessing the performance of the trained models (Fig. 5g), we are using the area under the receiver operating characteristic curve (AUROC) as a discrimination measure. Since the clinical diagnosis in case of PCa is based on the whole image, we are evaluating the results of the downstream task predictions as patient-wise AUC from the whole 3D images. For the patient-wise PCa prediction, we are taking the maximum value of the predicted lesion masks the same way as it is previously published in^26^ on the same data source (see Fig. 5h,i).

To be able to compare the performance of the trained models to radiologists’ interpretation, we also calculate the radiologists’ performance using the PI-RADS scores for the clinical index lesion as predictions and the maximum Gleason score of the systematic and targeted biopsy as the ground truth. According to PI-RADS, index lesions are scored on a Likert scale from 1 to 5 with higher scores indicating a higher risk of csPCa. The category of PI-RADS 3 has equivocal and PI-RADS 4 has high risk for csPCa^27^, which make these two categories the most informative area on the ROC curves. Thus, we calculate the sensitivity and specificity for PI-RADS $\ge$ 4 and PI-RADS $\ge$ 3. Calculating the performance of the radiologists during clinical practice provides a fixed reference point.

### Training protocol

The 625 data records were split into a training and hold out independent test set by stratifying exams by institution, PROSTATEx or local, and by the prevalence of csPC. The training set was used for 5-fold cross-validation. The exact distribution can be seen in Table 3. As there is an imbalance in the dataset, a balanced data loader is applied during the training.

**Table 3:** Patient distribution in the training and in the test set regarding csPCa.

Exams	Without csPCa	With csPCa	Sum
Training set	327	169	496
Test set	83	46	129
Sum	410	215	625

Small prostate lesion sizes compared to the size of the entire image introduce significant noise during the training^17^, especially as we have a limited number of exams with csPCa. Therefore, we are performing the same prostate-cropping mechanism based on the form of the prostate as Netzer et al.^26^ to increase the training stability.

The images are preprocessed by the automated image preprocessing algorithm of nnU-Net^25^. Specifically, the image modalities were linearly resampled to a common resolution with the spacing of 0.3125 mm in the transverse plane. The slice distance of 3 mm on the longitudinal axis was common in both datasets and remained unchanged. In addition to the spatial resampling, the T2-weighted and DWIs with high B-value were normalized patient-wise, but the ADC maps were normalized by intensity statistics across the training dataset as the voxel intensities can be ordered into a physical quantity. The resulting input patch size was 320x256x20 in the x-y-z axes, respectively.

Compared to the standard nnU-Net settings, we implemented a balanced sampling of patients regarding the prevalence of csPC. We used Mish activation function instead of Leaky ReLU, Ranger instead of SGD optimizer, a cosine anneal instead of Poly learning rate scheduler, and an initial learning rate of 0.001 instead of 0.01 following Netzer et al.^26^, which resulted in more stable training and better validation results.

We trained the 3D nnU-Net instance—consisting of 5 models—for each configuration of different dataset preprocessing techniques (see section “Registration methods and annotations for training”) and the use of misalignment data augmentation (see section “Misalignment augmentation”). We optimized the instances using early stopping and tuned the misalignment augmentation probability with respect to the 5-fold-cross-validation AUROC. To reduce the hyperparameter space for the probability of misalignment augmentation, we applied misalignment augmentation with the probabilities of $P=\left\{ 0.0, 0.1, 0.2, 0.4\right\}$ for the transformations to occur at all. The cross-validation results can be seen in ‘Supplementary Table S3’. The final models are then ensembled and evaluated on the independent test set using bootstrapping with 1000 replications to provide 95% confidence intervals. Additionally, we calculate p-values using the DeLong test^58^ to determine the statistical significance of the performance increase. We consider differences in the performance with $p<0.05$ statistically significant.

### Supplementary Information

Supplementary Tables.

## Data Availability

MRI exams from the PROSTATEx challenge^37^ are available at https://doi.org/10.7937/K9TCIA.2017.MURS5CL. MRI exams from our in-house cohort can not be made publicly available, due to data protection requirements. We make our proposed misalignment augmentations publicly available as part of the batchgenerators framework^52^ https://github.com/MIC-DKFZ/batchgenerators and integrate it into a nnU-Net^52^ trainer https://github.com/MIC-DKFZ/misalignment_DA.
