# Supplementary material for: Addressing image misalignments in multi-parametric prostate MRI for enhanced computer-aided diagnosis of prostate cancer
Source: Sci Rep. 2023 Nov 13;13:19805. doi: 10.1038/s41598-023-46747-z (PMC10643562; doi:10.1038/s41598-023-46747-z)
Supplement: Supplementary file 1 — Supplementary Tables. [file 41598_2023_46747_MOESM1_ESM.pdf]

Supplementary Material

Addressing image misalignments in multi-parametric prostate MRI for enhanced computer-aided diagnosis of prostate cancer

Balint Kovacs[1,2,3,@], **Nils Netzer**[2,3], **Michael Baumgartner**[1,4,5], **Adrian Schrader**[2,3], **Fabian Isensee**[1,4], **Cedric Weißer**[2,3], **Ivo Wolf**[6], **Magdalena Görtz**[7,8], **Paul F. Jaeger**[4,9], **Victoria Schütz**[8], **Ralf Floca**[1], **Regula Gnirs**[2], **Albrecht Stenzinger**[10], **Markus Hohenfellner**[8], **Heinz-Peter Schlemmer**[2,11], **David Bonekamp**[2,3,11,μ], **and Klaus H. Maier-Hein**[1,4,11,12,μ]

[1]Division of Medical Image Computing, German Cancer Research Center (DKFZ) Heidelberg, Heidelberg, Germany
[2]Division of Radiology, German Cancer Research Center (DKFZ) Heidelberg, Heidelberg, Germany
[3]Medical Faculty Heidelberg, Heidelberg University, Heidelberg, Germany
[4]Helmholtz Imaging, German Cancer Research Center (DKFZ) Heidelberg, Heidelberg, Germany
[5]Faculty of Mathematics and Computer Science, Heidelberg University, Heidelberg, Germany
[6]Mannheim University of Applied Sciences, Mannheim, Germany
[7]Junior Clinical Cooperation Unit 'Multiparametric methods for early detection of prostate cancer', German Cancer Research Center (DKFZ) Heidelberg, Heidelberg, Germany
[8]Department of Urology, University of Heidelberg Medical Center, Heidelberg, Germany
[9]Interactive Machine Learning Group, German Cancer Research Center (DKFZ) Heidelberg, Heidelberg, Germany
[10]Institute of Pathology, University of Heidelberg Medical Center, Heidelberg, Germany
[11]German Cancer Consortium (DKTK), DKFZ, core center Heidelberg, Germany
[12]Pattern Analysis and Learning Group, Department of Radiation Oncology, Heidelberg University Hospital, Heidelberg, Germany
[μ]These authors contributed equally to this work
[@]Corresponding author
E-mail address: balint.kovacs@dkfz-heidelberg.de
Postal address: Im Neuenheimer Feld 223, 69120 Heidelberg, Germany

S1 In-house dataset details

Supplementary Table S1. Demographic and clinical characteristics of the in-house dataset.

Characteristic	Training set	Test set
No. exams	335	86
• without csPCa	222 (66.3 %)	54 (62.8 %)
• with csPCa	113 (33.7 %)	32 (37.2 %)
Median age (years)	64	64
Mean gland volume(mL)	54.26	59.76
Mean PSA (ng/mL)	8.79	10.05
MRI scanners		
• Trio Tim	3	2
• Biograph	5	2
• Aera	7	1
• Prisma	320	81
Exams without MRI-detected lesion	13 (3.9 %)	4 (4.7 %)
MRI-detected index lesions		
• PI-RADS 2	43 (12.8 %)	10 (11.6 %)
• PI-RADS 3	83 (24.8 %)	19 (22.1 %)
• PI-RADS 4	129 (38.5 %)	34 (39.5 %)
• PI-RADS 5	67 (20.0 %)	19 (22.1 %)
No. csPCa/patient		
• 1 lesion	77	21
• 2 lesions	33	8
• 3 lesions	3	3
csPCa location		
• Peripheral zone	102	34
• Transition zone	42	12
• Multi zone	8	0
ISUP Grade		
• no PCa	148 (44.2 %)	36 (41.9 %)
• Gleason 3+3	74 (22.1 %)	18 (20.9 %)
• ISUP 2	63 (18.8 %)	22 (25.6 %)
• ISUP 3	21 (6.3 %)	3 (3.5 %)
• ISUP 4	12 (3.6 %)	2 (2.3 %)
• ISUP 5	17 (5.1 %)	5 (5.8 %)

S2 Distribution of the csPCa lesion(s) through the cohorts

Supplementary Table S2. Exam distribution through the datasets with respect to the number of csPCa lesions.

Exams	1 lesion	2 lesions	3 lesions
PROSTATEx	65	4	1
In-house dataset	98	41	6
Sum	163	45	7

S3 5-Fold Cross-Validation Results

Supplementary Table S3. AUROC results of the 5-fold cross-validation

5-fold cross-validation AUROC		Without registration	B-spline registration	GT-matching (reference)
Misalignment augm.	P = 0.0	78.04 %	79.19 %	80.31 %
	P = 0.1	80.58 %	81.10 %	82.52 %
	P = 0.2	80.67 %	80.03 %	81.60 %
	P = 0.4	80.21 %	79.95 %	80.75 %

S4 Test results across multiple centers

Supplementary Table S4. AUROC results on the test set across multiple centers

AUROC (test set)		Unregistered dataset	Registered dataset
without misalignment augm.	PROSTATEx	78.33 %	80.54 %
	in-house	75.49 %	77.43 %
with misalignment augm.	PROSTATEx	85.59 %	86.70 %
	in-house	77.78 %	79.98 %
